# Potential of cannabidiol as acne and acne scar treatment: novel insights into molecular pathways of pathophysiological factors

**DOI:** 10.1007/s00403-024-03131-9

**Published:** 2024-06-21

**Authors:** Jun Hyo Lee, Ji Young Yoon, Dong Hyo Kim, Yoon Gyung Kwon, Geun-Hyeong Kim, Byoung Jun Park, Dae Hun Suh

**Affiliations:** 1 Department of Dermatology, Seoul National University College of Medicine, 101 Daehak-ro, Jongno-gu, Seoul, 03080 Republic of Korea; 2 Acne, Rosacea, Seborrheic Dermatitis and Hidradenitis Suppurativa Research Laboratory, Seoul National University Hospital, Seoul, 03080 Republic of Korea; 3 Skin and Natural Products Laboratory, Kolmar Korea Co., Ltd, Seoul, Republic of Korea

**Keywords:** Acne, Cannabidiol, Inflammation, Keratinocytes, Lipid modulation, Sebocytes

## Abstract

Cannabidiol (CBD), which is derived from hemp, is gaining recognition because of its anti-inflammatory and lipid-modulating properties that could be utilized to treat acne. We conducted experiments to quantitatively assess the effects of CBD on acne-related cellular pathways. SEB-1 sebocytes and HaCaT keratinocytes were exposed to various CBD concentrations. CBD exhibited a concentration-dependent impact on cell viability and notably reduced SEB-1 viability; furthermore, it induced apoptosis and a significant increase in the apoptotic area at higher concentrations. Additionally, CBD remarkably reduced pro-inflammatory cytokines, including CXCL8, IL-1α, and IL-1β. Additionally, it inhibited lipid synthesis by modulating the AMPK-SREBP-1 pathway and effectively reduced hyperkeratinization-related protein keratin 16. Simultaneously, CBD stimulated the synthesis of elastin, collagen 1, and collagen 3. These findings emphasize the potential of CBD for the management of acne because of its anti-inflammatory, apoptotic, and lipid-inhibitory effects. Notably, the modulation of the Akt/AMPK-SREBP-1 pathway revealed a novel and promising mechanism that could address the pathogenesis of acne.

## Introduction

Acne is a highly prevalent disease with a peak incidence during the teenage years and the potential to persist into the third decade of life; furthermore, it ranks as the eighth most prevalent disease worldwide [1, 2]. Although the exact cause of acne remains unknown, it is believed to result from a combination of several factors rather than a single cause. The pathophysiology of acne can be broadly categorized into four factors, abnormal follicular keratinization, increased sebum production, the inflammatory response, and the presence and activity of *Cutibacterium acnes* bacteria, which are considered significant contributors to this condition [3]. *C. acnes* colonizes at the pilosebaceous units, specifically in regions densely populated with sebaceous follicles, which create a lipid-rich environment that serves as a pathological hallmark of this skin condition [4]. Various treatments have been attempted for active acne lesions and scars, but their effectiveness has been disappointing, resulting in significantly higher levels of perceived stigmatization for individuals with acne than for those with healthy skin [5].

**Fig. 1:**
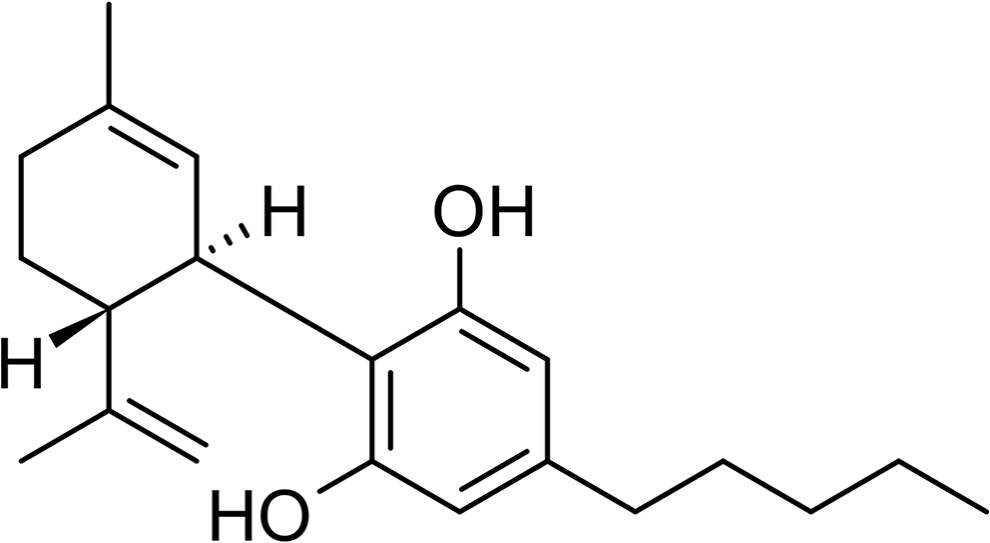
Chemical structure of CBD

In 2019, the Ministry of Food and Drug Safety of Korea implemented the “Amendment to the Narcotic Control Act,” which permitted the domestic importation of hemp-based drugs licensed abroad, including cannabidiol (CBD), which is the main nonpsychotropic substance found in hemp (Fig. 1). This progressive move was aimed at broadening treatment opportunities for rare and intractable diseases in Korea. CBD has demonstrated dose-dependent inhibition of excessive lipid synthesis and anti-proliferative and anti-inflammatory effects on sebaceous gland cells [6]. In another study involving human keratinocyte cell lines, the application of a 0.6% hemp seed hexane extract solution demonstrated a reduction in *C. acnes*-induced inflammation by suppressing pro-inflammatory cytokines, specifically IL-1β and IL-8 [7]. Additionally, CBD has been shown to inhibit keratinocyte hyperproliferation in vitro through multiple receptors [8]. In vivo experiments have also shown promising results. During a 12-week split-body trial involving patients with psoriasis, the use of topical 2.5% CBD ointment and placebo twice daily resulted in significant improvements, especially in scaling, without any major adverse effects [9]. Therefore, CBD could be an effective treatment for acne by targeting potential pathophysiological mechanisms.

To advance our understanding of the effects of CBD on cell lines, this study aimed to examine the potential of CBD to treat and prevent acne by exploring specific pathways, such as the intracellular inflammatory response and triglyceride reduction, in sebocytes. Additionally, this study sought to introduce a fresh approach to acne treatment and move beyond conventional methods. Through comprehensive analyses, this innovative approach could provide more effective treatment options, particularly for patients susceptible to inflammation and scarring.

## Materials and methods

### Test material and culture

#### CBD

CBD was prepared at concentrations of 0.1, 1, 5, 10, 50, and 100 µM (Kolmar Korea Co., Ltd, Seoul, Republic of Korea).

#### Cell culture and preparation

The SEB-1 immortalized human sebocyte cell line, which is a transformed cell line generated by transfection with the SV40 large T antigen, has been mentioned in many reports of acne-related studies [10, 11]. Passage 28 SEB-1 immortalized human sebocytes were used in all experiments. The standard culture medium for growing SEB-1 cells consisted of 5.5 mM glucose supplemented with Ham’s F-12 (3:1) (Invitrogen, Carlsbad, CA, USA), 2.5% fetal bovine serum (HyClone, Logan, UT, USA), adenine (180 µM) (Sigma, St. Louis, MO, USA), hydrocortisone (0.4 µg/ml), insulin (10 ng/ml), epidermal growth factor (3 ng/ml) (Austral Biologicals, San Ramon, CA, USA), and cholera toxin (0.12 nM) (Sigma). Serum-free sebocyte medium used in lipogenesis experiments consisted of DMEM containing 5.5mM glucose and antibiotics without any other additives [12].

The Detroit 551 cell (human embryonic skin fibroblast cell line, ATCC CCL-110) was maintained in Eagle’s minimum essential medium (EMEM) and Dulbecco’s modified Eagle’s medium (Sigma, St. Louis, MO, USA) plus 10% fetal bovine serum (FBS) (Invitrogen, San Diego, CA, USA), at 37 °C and in a humidified atmosphere containing 5% CO2 [13]. The HaCaT cell (immortalized human keratinocyte line) was cultured as monolayers in a standard culture medium (DMEM supplemented with 5% fetal calf serum, 2 mM glutamine, 100 IU/ml penicillin) at 37 °C in a humidified atmosphere containing 5% CO_2_.

### Cytotoxicity analysis

SEB-1 cells were treated with different concentrations of CBD and cultured for 24 h, 48 h, and 72 h. After the respective incubation periods, 10 µl of CCK8 solution (Dojindo, Rockville, MD, USA) was added to each well. After a 1-hour reaction at 37 °C, cytotoxicity was assessed by measuring absorbance at 450 nm using a spectrophotometer. To evaluate the potential cytotoxic effects of CBD on other cell lines, HaCaT cells were subjected to similar treatments, and their respective cytotoxicity levels were determined. ApopTag peroxidase in situ apoptosis detection (cell TUNEL stain) was used to assess whether apoptosis was induced.

### Inhibitory concentration of CBD on *C. acnes*

*C. acnes* (ATCC 6919), the causative microorganism of acne, was purchased from the Korea Microbial Strains Bank (Seoul, Republic of Korea). These strains were cultured in reinforced clostridium medium (Becton Dickinson, Franklin Lakes, NJ, USA) at 37 °C under anaerobic conditions. Before using the strains in the experiments, *C. acnes* was cultured in tryptic soy broth (BD) medium supplemented with 5% (volume/volume) defibrinated sheep blood at 37 °C until reaching an OD600 of 1.0 (logarithmic growth phase). The strains that reached the log value were filtered after centrifugation at 5,000 g and 4 °C for 15 min; then, they were heat killed at 95 °C for 20 min before use. *C. acnes* strains at a concentration of 2 × 10^7^ CFU/ml were prepared in liquid medium. Different concentrations of CBD were added to 96-well plates. Then, plates were incubated with *C. acnes* strains for 48 to 72 h under anaerobic conditions. The suppression of *C. acnes* was confirmed by assessing the reduction of *C. acnes* using absorbance measurements.

### Inflammatory cytokine analysis

To confirm the anti-inflammatory effect of CBD, an inflammatory cytokine quantitative PCR was performed after inducing inflammation in SEB-1 cells by adding *C. acnes* or lipopolysaccharide (LPS). The analysis was conducted using a real-time PCR and a 384-well instrument (LightCycler® 480; Roche, Indianapolis, IN, USA) with SYBR® Green quantitative PCR Master (Applied Biosystems, Foster City, CA, USA) for GAPDH, TNF-α, IL-1α, IL-1β, IL-8, and IL-6.

### Lipogenesis analysis

After treating SEB-1 sebocytes with CBD at concentrations of 5 and 10 µM for 48 h, proteins and pathways involved in lipid synthesis were evaluated using a Western blot analysis. To understand the potential mediators of CBD-induced SREBP-1 inhibition, AKT and AMPK, which are both known to regulate SREBP-1, were studied [14, 15]. The antibodies used included β-actin mouse antibody (Santa Cruz Biotechnology, Santa Cruz, CA, USA), SREBP-1 rabbit antibody (Santa Cruz Biotechnology), phospho-AMPKα1/2 rabbit antibody (Abcam, Cambridge, MA, USA), and phospho-AKT Thr308 (Cell Signaling Technology, Beverly, MA, USA). HMG CoA synthase and HMG CoA reductase, which are related to the triglyceride subpathway, were analyzed by performing a real-time PCR with a 384-well instrument and SYBR® Green quantitative PCR Master as mentioned.

SEB-1 cells were seeded on four-well culture slides at 5 × 10^4^ cells per well. After 48 h, they were washed with phosphate-buffered saline and fixed with 10% formalin for 10 min. SEB-1 cells were stained using the NovaUltra Oil red O stain kit (IHC World, Woodstock, MD, USA) and observed under a microscope at 400× magnification to verify differences in lipogenesis.

Immunocytochemistry staining intensity was quantified using a fluorescence microscope with the NIS elements BR imaging system (version 4.30; Nikon, Minato, Japan), and an analysis was performed using the TINA densitometric program (Raytest Isoto-penmebgerate, Straubenhardt, Germany).

### Hyperkeratinization-related protein

The change in keratin 16 was assessed through western blot and mRNA analyses using keratin 16 rabbit antibody (Santa Cruz Biotechnology) after subjecting HaCaT cells to 0-, 5-, or 10-µM CBD treatment for 24 h. To ensure accurate protein quantification, the proportion of keratin 16 to β-actin was used for normalization, thereby maintaining consistency in protein loading across the gel.

### Scar formation-related protein synthesis of fibroblasts

The changes in collagen 1, collagen 3, and elastin in fibroblasts were assessed by performing a western blot analysis, mRNA analysis, and DAPI staining. Proteins were extracted using cell lysis buffer (Cell Signaling Technology), and their contents were determined using the BCA Protein Assay (Pierce, Rockford, IL, USA). Equal protein amounts were separated on 10% SDS-PAGE gels and transferred to a membrane. Antibodies against collagen 1, collagen 3, and elastin were applied after exposing the fibroblast cells to 0-, 1-, or 5-µM CBD treatment for 48 h. To ensure accurate quantification, ratios of protein to β-actin were used for normalization. Immunocytochemistry staining was performed by using the same method as that used for the lipogenesis analysis.

### Statistical analysis

The results are expressed as the mean and standard deviation of three independent experiments. A statistical significance test of the control group and experimental group was performed using SPSS (version 27.0; Armonk, NY, USA), and *P* < 0.05 was considered statistically significant. Data were evaluated using a one-way analysis of variance and GraphPad Prism (version 5.0) software to determine statistical significance between treatments.

## Results

### Assessment of the toxic effects of CBD on SEB-1 and HaCaT cells

**Fig. 2:**
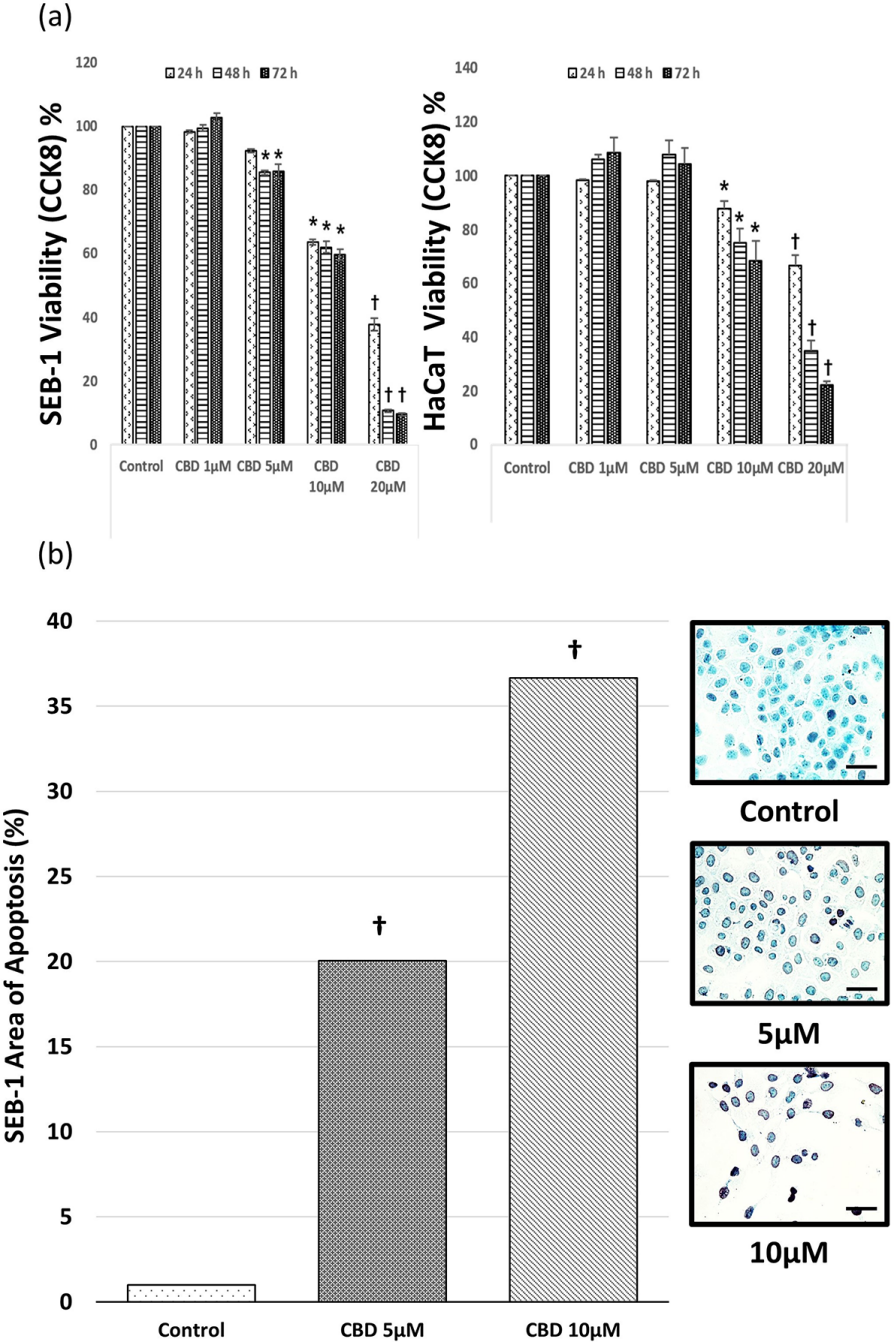
CBD induces apoptosis in SEB-1 cells and HaCaT cells. (**a**) SEB-1 cells and HaCaT cells were exposed to CBD at concentrations of 1, 5, 10 or 20 µM for 48 h, followed by confirmation of apoptosis using the TUNEL assay method. (**b**) Quantitative analysis of apoptosis staining was conducted and the results were graphed. Representative micrograph images of SEB-1 cells treated with CBD showing the induction of apoptosis (400X magnification). Data are presented from a sample size of *n* = 9 independent experiments. All are at the same magnification (bar = 50 μm). CBD, cannabidiol; TUNEL, terminal deoxynucleotidyl transferase dUTP nick end labeling. *, *P* < 0.05, †, *P* < 0.001

To investigate the temporal effects of CBD on the viability of SEB-1 and HaCaT cells, a CCK8 assay was performed (Fig. 2a). When treated with CBD, both SEB-1 and HaCaT cells exhibited a significant reduction in cellular growth. When subjected to a CBD concentration of 1 µM, SEB-1 cells had an average viability rate of 98.2%; therefore, the difference was not statistically significant. Viability rates of SEB-1 cells subjected to CBD concentrations of 5 µM, 10 µM, and 20 µM were 92.4%, 63.7%, and 37.7%, respectively, at 24 h. These rates further decreased to 85.5%, 62.1%, and 10.7%, respectively, at 48 h, and 85.9%, 59.8%, and 9.5%, respectively, at 72 h. The CBD concentration appeared to have a greater inhibitory effect on proliferation. HaCaT cells also exhibited a concentration-dependent response; however, they showed a higher threshold of inhibition with a CBD concentration of 10 µM. Furthermore, after 72 h, the rates of HaCaT cell viability after the application of CBD concentrations of 5 µM, 10 µM, and 20 µM were 104.5%, 68.4%, and 22.2%, respectively.

A TUNEL (terminal deoxynucleotidyl transferase dUTP nick end labeling) assay was performed to confirm apoptosis of SEB-1 cells treated for 48 h with CBD concentrations of 5 µM or 10 µM (Fig. 2b). At a concentration of 5 µM, the area of apoptosis was 20.05%, and it was 36.65% at a concentration of 10 µM; however, it was 1% for the control group (*P* < 0.001 compared to baseline, respectively).

### Effects of CBD on LPS-induced or ***C. acnes***-induced inflammation of SEB-1 cells

**Fig. 3:**
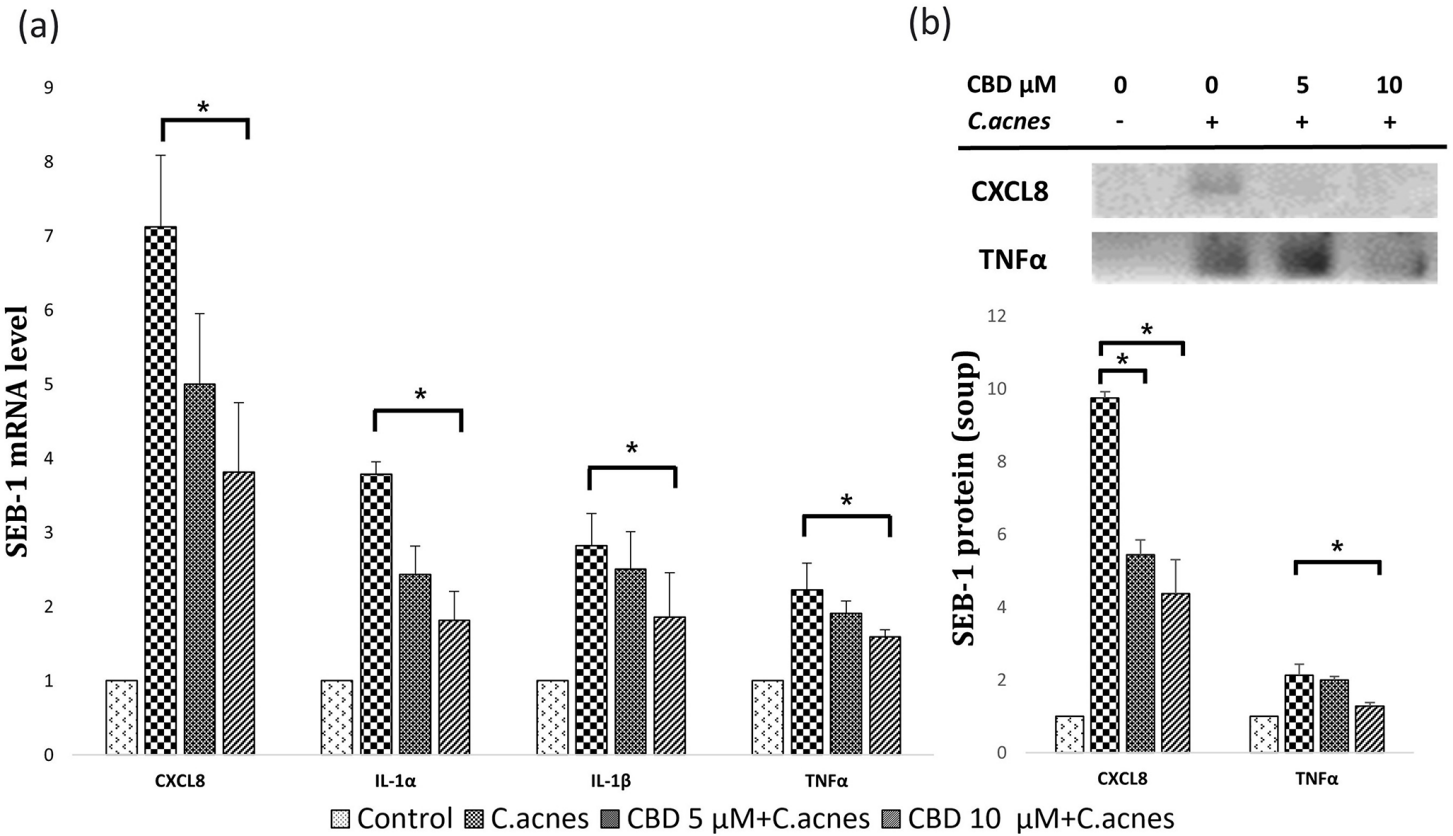
CBD modulates inflammatory cytokine expression in SEB-1 cells exposed to *C. acnes*. SEB-1 cells were treated with CBD at concentrations of 0 µM, 5 µM, and 10 µM in the presence of *C. acnes* to assess changes in inflammatory cytokines. (a) Quantitative real-time PCR validated reduced mRNA expression levels of CXCL8, IL-1α, IL-1β, and TNFα post-CBD treatment compared to the untreated control. (b) Western blot analysis quantified protein levels of CXCL8 and TNFα. Quantified data were graphically represented, showcasing decreased protein levels in response to CBD treatment. CBD, cannabidiol; *C. acnes*, *Cutibacterium acnes*. *, *P* < 0.05

CBD was treated in conjunction with *C. acnes* or LPS at concentrations of 0, 5, and 10 µM, followed by a 24-hour incubation period for the protein and mRNA analyses. Decreases observed in the mRNA levels of CXCL8, IL-1α, and IL-1β corresponded to the escalating concentrations of CBD (Fig. 3a). The relative level of CXCL8 increased to 7.12 after incubation with *C. acnes*. However, with the addition of 5 µM and 10 µM of CBD, the levels decreased to 5.00 and 3.81, respectively (*P* < 0.05 for both). Regarding IL-1α and IL-1β, the levels decreased from 3.79 to 2.44 and 1.82, 2.82 to 2.50 and 1.86 with CBD concentrations of 0, 5, and 10 µM (all *P* < 0.05). These reductions were consistently evident during the protein analysis and western blot analysis, in which both CXCL8 and TNF-α cytokines exhibited dose-dependent reductions with CBD treatment (Fig. 3b).

### Effects of CBD on lipogenesis via the AMPK-SREBP-1 signaling pathway in SEB-1 sebocytes

**Fig. 4:**
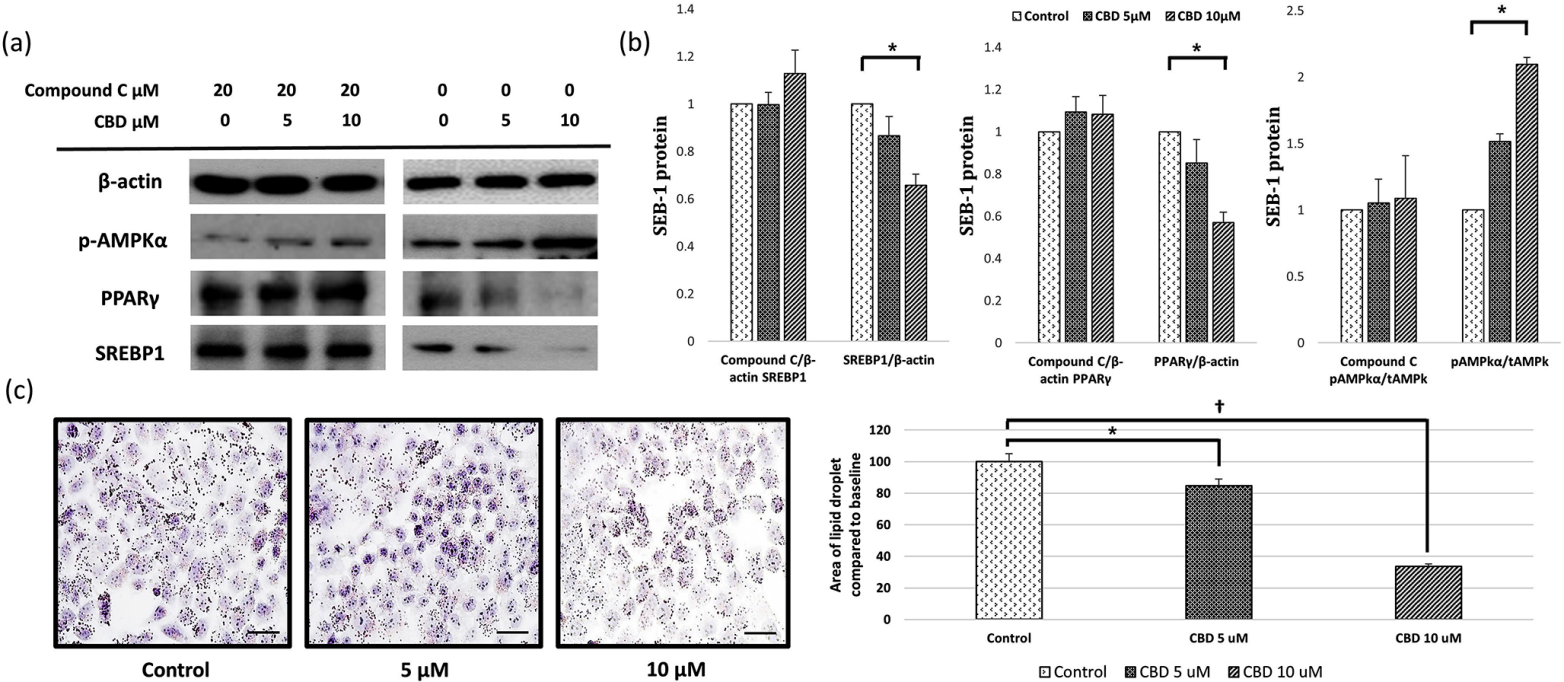
Effect of CBD on lipogenesis pathway in SEB-1 cells. CBD reduces SEB-1 lipogenesis by inhibiting the expression of sterol regulatory element binding protein (SREBP)-1 through the AMP-activated protein kinase (AMPK) pathway. (**a**) SEB-1 cells were pretreated by CBD with or without 20 µM of compound C for 24 h before western blot. Levels of phospho-/total AMPK, PPARγ, and SREBP1 were measured. (**b**) Western blot bands of SREBP-1/β-actin, PPARγ/β-actin, and phospho-/total AMPKα were quantified and presented in a graph. (**c**) Oil red staining analysis was performed and graphed (400X). All are at the same magnification (bar = 50 μm). CBD, cannabidiol; SREBP, sterol regulatory element binding protein; AMPK, AMP activated protein kinase; PPARγ, peroxisome proliferator-activated receptor gamma. *, *P* < 0.05; †, *P* < 0.001

The lipid-inhibitory effect of CBD was confirmed through the regulation of AMPK-SREBP-1 signaling using western blotting and quantitative polymerase chain reaction (PCR) methods. CBD activated AMPK and reduced SREBP, PPARγ, and lipid production in SEB-1 sebocytes (Fig. 4a). These findings revealed a significant decrease in SREBP-1 mRNA as CBD concentrations increased (0.87 and 0.66, respectively) compared to the baseline values with 5 and 10 µM CBD (*P* < 0.05) (Fig. 4b). This decrease was accompanied by a reduction in PPARγ and increase in p-AMPKα levels (0.85 and 0.57 for PPARγ and 1.52 and 2.10 for p-AMPKα, respectively) compared to the baseline levels with 5 and 10 µM CBD (all *P* < 0.05) (Fig. 4b). To validate the involvement of the AMPK pathway in this effect, treatment with an AMPK inhibitor, compound C, was conducted, which reversed the effect of CBD on SREBP-1, PPARγ, and p-AMPKα (1.00 and 1.13, 1.09 and 1.08, and 1.05 and 1.09, respectively) compared to the baseline values with 5 and 10 µM CBD (Fig. 4b). This suggests that CBD may decrease SREBP-1 by inhibiting PPARγ and increasing p-AMPKα, ultimately leading to a reduction in lipid synthesis in sebocytes through the Akt/AMPK-SREBP-1 signaling pathway. In SEB-1 sebocytes exposed to C. acnes, no significant increase in the expression of genes associated with lipogenesis was observed. However, similar to SEB-1 cells not exposed to C. acnes, exposure to CBD showed a trend of inhibiting gene expression.

To confirm the inhibition of lipogenesis, an Oil Red O staining analysis was performed to assess the area of red lipid droplets. The lipid droplet area decreased to 84.6% compared to the baseline values after 48 h with 5-µM CBD treatment, and it further decreased to 33.5% after 48 h with 10-µM CBD treatment, demonstrating concentration-dependent inhibition (*P* < 0.05 and *P* < 0.001, respectively) (Fig. 4c).

**Fig. 5:**
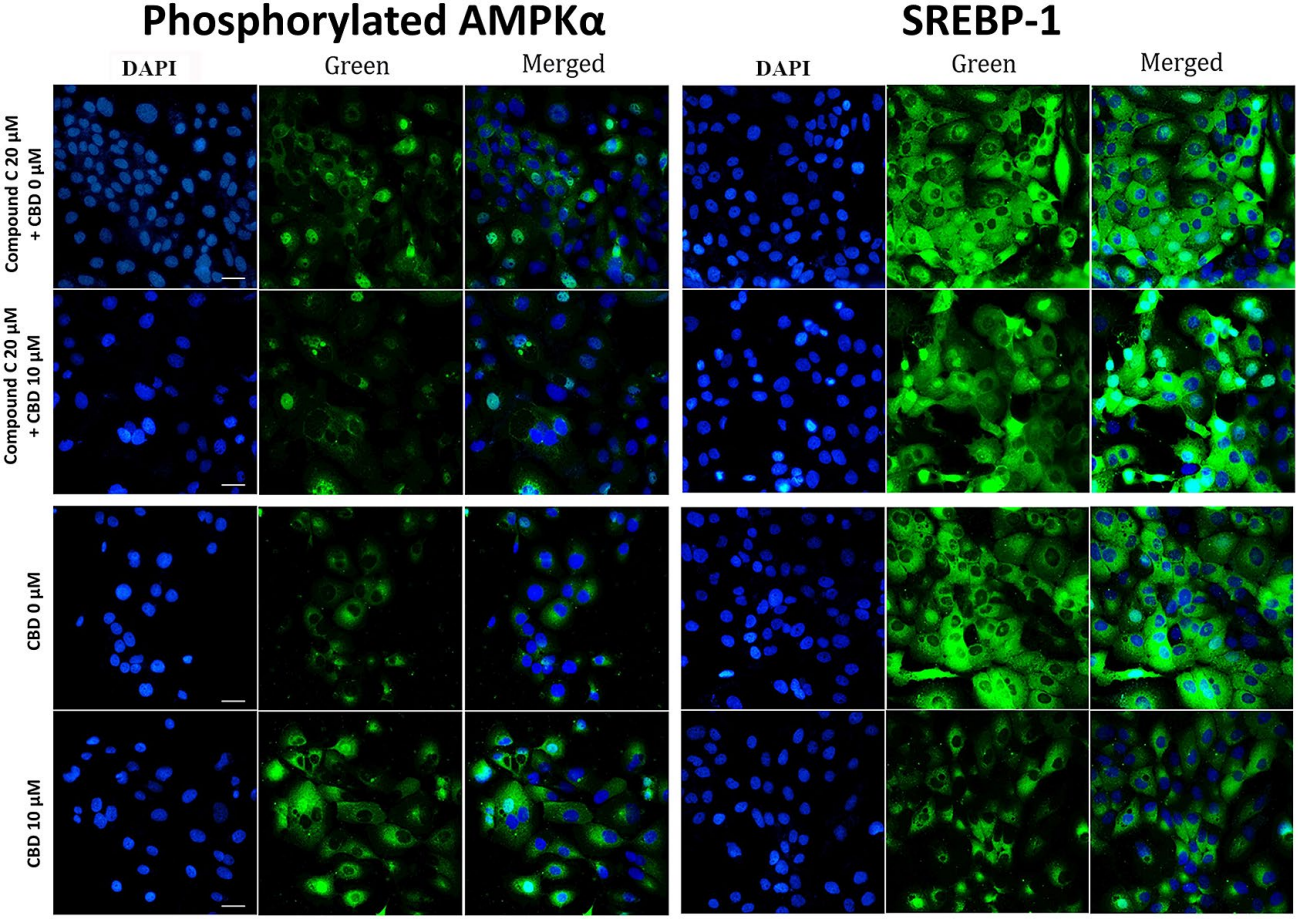
Representative DAPI staining images of CBD treated SEB-1 cells. Representative DAPI staining images of CBD treated SEB-1 cells. SEB-1 cells were treated with CBD at concentrations of 0 µM and 10 µM for a duration of 48 h. DAPI staining was performed to assess the impact of CBD treatment. Phosphorylated AMPKα expression and SREBP-1 expression were evaluated using DAPI staining (400X). All are at the same magnification (bar = 50 μm). CBD, cannabidiol; SREBP, sterol regulatory element binding protein; AMPK, AMP activated protein kinase; DAPI, 4’,6-diamidino-2-phenylindole

DAPI (4′,6-diamidino-2-phenylindole) staining has shown corresponding results, indicating that CBD increases p-AMPKα and inhibits SREBP-1, and that compound C was able to reverse this action (Fig. 5). The green fluorescence signals of p-AMPKα and SREBP-1 were quantified using ImageJ software. The signal intensity divided by the cell number per field of p-AMPKα was 0.52 when treated with 0 μM CBD, significantly increasing to 0.76 with 10 μM CBD treatment (*P*<0.05). However, this value decreased to 0.36 after subsequent treatment with 20 μM compound C, even lower than that of the baseline. For SREBP-1, the signal intensity divided by the cell number per field was 0.35 upon treatment with 0 μM CBD, but significantly dropped to 0.26 with 10 μM CBD treatment (*P*<0.05). Treatment with 20 μM compound C with CBD 10 μM showed a signal intensity of 0.61, indicating the reversal of CBD’s effects, even higher than default value.

### Effects of CBD on hyperkeratinazation-related protein keratin 16 using HaCaT cells

**Fig. 6:**
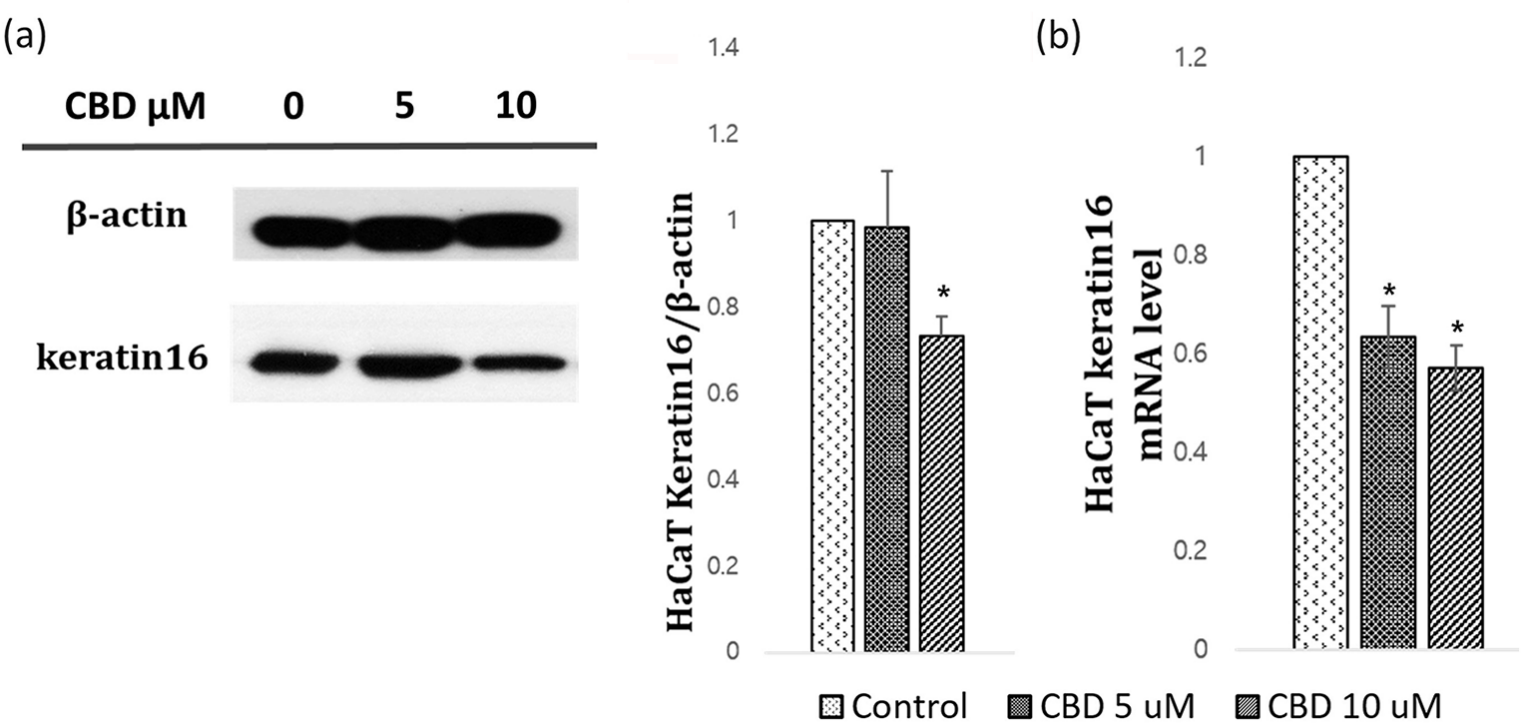
Inhibition of hyperkeratinization by CBD treatment in HaCaT cells. HaCaT cells were subjected to CBD treatment at concentrations of 0 µM, 5 µM, and 10 µM for a duration of 24 h. (**a**) Western blot analysis was employed to validate the reduction in keratin 16 levels. (**b**) Alterations in the mRNA expression of keratin 16 were verified through real-time PCR. CBD, cannabidiol. *, *P* < 0.05

HaCaT cells were treated with CBD concentrations of 0, 5, and 10 μM and underwent western blot and quantitative real-time mRNA analyses to assess alterations in hyperkeratinization-related protein keratin 16. The western blot analysis revealed that keratin 16 decreased to 0.99 and 0.73 after 5- and 10-μM CBD treatments compared to baseline values (*P* = 0.89 and *P* = 0.01, respectively) (Fig. 6a). In line with the western blot analysis results, the quantitative real-time method also indicated a decrease in the keratin 16 levels. Specifically, after 5- and 10-μM CBD treatment, the keratin 16 levels decreased to 0.63 and 0.58, respectively (*P*<0.05) (Fig. 6b). These results confirm the inhibitory effect of CBD on hyperkeratinization.

### Effects of CBD on scar formation-related protein synthesis of fibroblasts

**Fig. 7:**
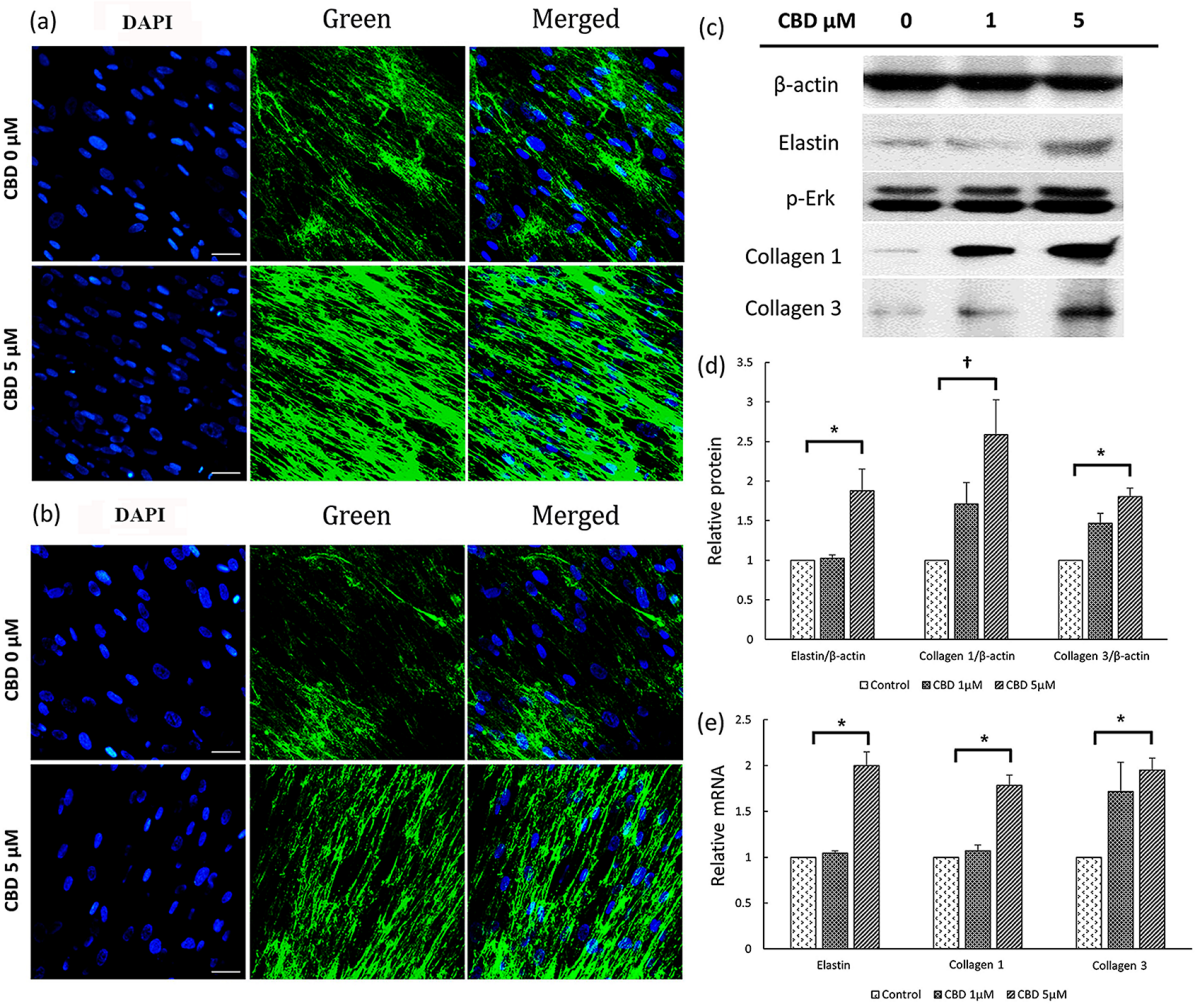
Changes in the expression of proteins related to scar formation induced by CBD. (**a**, **b**) Fibroblasts were treated with CBD at concentrations of 0 µM and 5 µM for a duration of 48 h. DAPI staining was performed to assess the impact of CBD treatment. Collagen 1 (a) and collagen 3 (b) expression were evaluated using DAPI staining (400×). All are at the same magnification (bar = 50 μm). (**c**) Western blot analysis confirmed changes in the expression of elastin, collagen 1, collagen 3, and p-Erk at CBD concentration of 1 µM or 5 µM. (**d**) Quantification of western blot bands for elastin/β-actin, collagen 1/β-actin, and collagen 3/β-actin was performed. (**e**) Quantitative real-time analysis confirmed the mRNA expression levels of elastin, collagen 1, and collagen 3. CBD, cannabidiol; DAPI, 4’,6-diamidino-2-phenylindole; p-ERK, phosphorylated ERK. *, *P* < 0.05; †, *P* < 0.00

DAPI staining was performed to investigate alterations in collagen 1 and collagen 3 synthesis in fibroblasts (Fig. 7a and b). Upon exposure to CBD at a concentration of 5 µM, the synthesis of both collagen 1 and collagen 3 increased significantly, as evident from the merged images. Subsequently, fibroblasts were subjected to 0, 1, and 5 µM of CBD for further analyses using western blot and quantitative real-time mRNA assessments to evaluate changes in scar-related proteins. At a CBD concentration of 1 µM, the relative quantities of elastin, collagen 1, and collagen 3 exhibited minor changes measuring 1.02, 1.71, and 1.47, respectively, in comparison to the baseline value of 1.00; however, these changes were not statistically significant (*P* = 0.40, *P* = 0.11, and *P* = 0.87, respectively) (Fig. 7c and d). In contrast, when exposed to a higher CBD concentration of 5 µM, the values significantly increased to 1.88, 2.59, and 1.80 (*P* < 0.05, *P* < 0.001, and *P* < 0.05, respectively) (Fig. 7c and d). The quantitative real-time analysis yielded consistent findings, with 5-µM CBD treatment resulting in mRNA levels of elastin, collagen 1, and collagen 3 reaching 2.00, 1.78, and 1.95, respectively (all *P* < 0.05) (Fig. 7e).

## Discussion

Acne, a prevalent dermatological condition with multifactorial origins, remains a challenging concern in both adolescent and adult populations [16]. CBD demonstrated effects on sebum production, inflammation, apoptosis, and hyperkeratinization, which underscore its potential to address multiple pathophysiological aspects of acne. By quantitatively assessing the impact of CBD on sebocyte viability, this study establishes a dose-dependent reduction, emphasizing CBD’s anti-proliferative properties. Moreover, the induction of apoptosis suggests a potential avenue to curtail hyperproliferation. The ability of CBD to counteract the *C. acnes*-induced inflammatory response by downregulating cytokines like CXCL8, IL-1α, and IL-1β further positions it as an anti-inflammatory agent for acne.

One of the study’s notable findings is CBD’s modulation of lipid synthesis through the Akt/AMPK-SREBP-1 pathway. This unique lipid-modulating effect is significant, as sebum overproduction is a hallmark of acne and contributes to pore blockages. Previous study on cultured human sebocytes demonstrated that activating transient receptor potential vanilloid-4 (TRPV4) ion channels resulted in the downregulation of nuclear receptor interacting protein-1 (NRIP1), which influences glucose and lipid metabolism [6]. While there isn’t a direct and well-established connection between the Akt/AMPK-SREBP-1 pathway and the TRPV4 ion channel, both pathways can influence cellular processes related to lipid metabolism and inflammation [17, 18]. The Akt/AMPK-SREBP-1 pathway primarily regulates lipid synthesis and metabolism within cells, while the TRPV4 ion channel has been associated with various cellular functions, including calcium signaling and regulation of cellular responses to mechanical and osmotic stimuli [19]. It’s possible that these pathways could indirectly interact or influence each other through broader cellular signaling networks. For instance, TRPV4 activation might impact cellular responses that intersect with the Akt/AMPK-SREBP-1 pathway, potentially influencing lipid metabolism or inflammation. However, such interactions would likely be complex and might involve intermediary signaling molecules or pathways that have yet to be fully elucidated. In parallel investigations, CBD treatment at 10 µM within an LPS-induced acne-like context exhibited decreased levels of TNF-α, IL-1β, and IL-6 [6].

Additionally, a separate in vitro study employing hemp seed hexane extracts (HSHE) demonstrated that the application of 0.6% HSHE to a human keratinocyte cell line led to a reduction in inflammation induced by *C. acnes* [7]. Notably, this effect was achieved through the down-regulation of pro-inflammatory cytokines, specifically IL-1β and IL-8. In a human study evaluating the safety and efficacy of cannabis seed extracts for acne, 3% cannabis seed extracts were found to reduce inflammation-induced erythema of the skin in a 12 week study and was found to be safe, well tolerated, non-allergenic, and non-irritating in the patient volunteers [20]. Finally, in UV-irradiated keratinocytes, CBD notably augmented the efficacy of antioxidant enzymes like superoxide dismutase and thioredoxin reductase [21]. This enhancement could offer potential benefits for individuals with acne, as a majority of the lesions manifest on sun-exposed regions of the skin. Collectively, these studies, along with our own findings, offer a coherent narrative of the potential for botanical extracts, such as hemp and cannabis seed extracts, to modulate inflammatory responses associated with acne.

There are several potential reasons why keratinocytes exhibit greater resistance in CBD-mediated inhibition experiments compared to sebocytes. A study showed that CBD was able to penetrate human keratinocytes and balance the oxidative stress response resulting from UVB irradiation and hydrogen peroxide [22]. This suggests that keratinocytes may be more resistant to CBD-induced inhibition because they are better able to absorb and utilize CBD. Another possible reason may be variations in the expression and density of cannabinoid receptors, such as CB1 and CB2 receptors between sebocytes and keratinocytes. Keratinocytes form the predominant component of the epidermis, serving as a protective barrier against external stressors. This characteristic might confer inherent resilience to external agents like CBD.

Presently, the primary approaches to address acne scars involve procedural methods such as subcision, chemical peels, and laser treatments, but their effectiveness is not consistently satisfactory [23, 24]. In a recent study, the use of topical epidermal growth factor ointment was found to stimulate elastin, collagen 1, and collagen 3 production within acne scars, leading to clinical improvements. Moreover, this study noted a reduction in IL-1 alpha and keratin 16, suggesting that CBD may have potential in enhancing the clinical outcomes of acne scarring [25]. In another study investigating CBD’s anti-wrinkle properties, UV-damaged fibroblasts exposed to 4 µM CBD exhibited a significant 27.7% increase in collagen content [26]. Although this concentration may seem similar to the 5 µM concentration discussed in this paper, it’s crucial to consider that the increase in collagen content showed notable distinctions. This variance might be linked to the UV-damaged condition of the cells, which could potentially hinder collagen synthesis. In addition to its known effect in inhibiting the action of matrix metalloproteinases, preventing extracellular matrix (ECM) degradation, CBD appeared to promote ECM generation at the mRNA level, demonstrating the potential for treatment approaches rather than procedures for acne scarring [27].

It is imperative to acknowledge the limitations of this study. While the cellular level investigations provide valuable mechanistic insights, clinical trials are necessary to ascertain the real-world efficacy and safety of CBD-based treatments. Ethical and legal considerations surrounding CBD usage should also be taken into account, especially given variations in regulations across regions. Future research avenues include well designed clinical trials to validate the efficacy of CBD-based therapies, alongside investigations into potential adverse effects.

In conclusion, this study highlights CBD’s potential to address multiple facets of acne pathophysiology through its anti-inflammatory, apoptotic, lipid-inhibitory effects, and modulation of the Akt/AMPK-SREBP-1 pathway. Additionally, it suggests the potential for CBD to contribute to the improvement of acne scarring through the synthesis of collagen and elastin. These findings offer a fresh perspective on acne management, suggesting that CBD-based treatments could provide a more comprehensive approach for individuals prone to inflammation and scarring. While further research is warranted, CBD’s unique mechanism of action presents a promising avenue for advancing acne therapeutics and improving patients’ quality of life.

## Data Availability

The data presented in this study are available on request from the corresponding author.

## Declarations

### Statement on any prior presentation

Contents of the manuscript have not been previously published and are not currently submitted elsewhere.
